# Ischaemic heart disease is the factor associated with severe COVID-19 in the urban population of Uzbekistan: a single‑center retrospective study

**DOI:** 10.1186/s12879-026-12798-6

**Published:** 2026-02-12

**Authors:** Nargiz Ibadullaeva, Erkin Musabaev, Aziza Khikmatullaeva, Leonid Padyukov

**Affiliations:** 1 Research Institute of Virology, Tashkent, Uzbekistan; 2 Division of Rheumatology, Department of Medicine Solna, Karolinska Institutet and Karolinska University Hospital, Stockholm, Sweden; 3 Center for Molecular Medicine, Karolinska Institutet, Stockholm, Sweden

**Keywords:** COVID-19, Severe infection, Ischaemic heart disease, ACE gene polymorphism

## Abstract

**Background:**

The course of disease development during the coronavirus disease 2019 **(**COVID-19) pandemic has demonstrated a very wide spectrum, with the most vulnerable group of severe disease comprising > 10% of cases worldwide. Previously, several clinical and laboratory phenotypes have been suggested for the prediction of severe disease courses with different impacts in diverse populations.

**Methods:**

Using a logistic regression model, we performed a study of 227 patients (37% with severe disease), all of whom were ethnically Uzbek, to identify predisease clinical phenotypes associated with disease severity, such as type 2 diabetes (T2D), obesity, hypertension and ischaemic heart disease (IHD), and ascertained the contribution of the angiotensin converting enzyme-encoding gene insertion/deletion (ACE I/D) rs1799752 and the interleukin-28 isoform B (IL28B) gene rs12979860 genetic markers.

**Results:**

We found that the greatest contribution to the severe disease group from IHD was observed before the start of infection, whereas the contributions of T2D and obesity were only nominally important for the model. Interestingly, the ACE rs1799752 DD genotype together with clinical phenotypes contributed to the discrimination of the severe disease group, but we detected no effect of the IL28B polymorphism. However, without the inclusion of clinical phenotypes in the model, we did not observe a significant ACE polymorphism association with COVID-19 severity (likelihood ratio test *p* = 0.1). We critically reviewed allelic frequencies for ACE rs1799752 in different populations and studies in an attempt to explain possible discrepancies in previously reported associations in diverse populations.

**Conclusions:**

In a modest group of patients from the Uzbek population, we confirmed the importance of IHD, metabolic disorders and ACE genetics in the development of severe COVID-19 infection in this population.

**Clinical trial number:**

Not applicable.

**Supplementary Information:**

The online version contains supplementary material available at 10.1186/s12879-026-12798-6.

## Introduction

Despite the implementation of measures to control coronavirus disease 2019 (COVID-19) and ongoing global vaccination efforts, cases persist due to the emergence of new severe acute respiratory syndrome coronavirus 2 (SARS-CoV-2) variants. The course of COVID-19 varies from asymptomatic and mild to severe/extremely severe forms that differ in terms of treatment decisions and health care strategies. Research has shown that factors related to the virus play crucial roles in COVID-19 outcomes [1–3]. Additionally, host factors, including age, comorbidities, and genetic polymorphisms, may influence disease risk, clinical manifestations, and outcomes [4–6].

The investigation of host factors, including gene polymorphisms, is crucial in infectious disease studies. Although genetic variations in infectious diseases are not causative, they may play a significant role in the predisposition to infection and disease course. They can also modify the contribution of other risk factors to the disease and interact with increasing disease risk or severity with age, exposure to environmental and occupational factors, ethnic habits, lifestyle behaviours, socioeconomic and ecological conditions, and type and access to treatment. Previous studies identified genetic determinants related to COVID-19, including the associations of genetic markers with susceptibility to infection and disease severity. The angiotensin converting enzyme-encoding gene insertion/deletion (ACE I/D) and interleukin-28 isoform B (IL28B) rs12979860 polymorphisms has previously been shown to be associated with the course of COVID-19 [7, 8]. However, these findings remain controversial and are often based on heterogeneous study designs with small sample sizes in diverse populations.

Allele and genotype frequencies in various ethnic populations may significantly differ, making genetic association studies in different ethnic groups essential for understanding risk factors and developing personalized medical approaches and treatments. These differences can significantly affect statistical models when testing clinical data and should be carefully assessed. Both for the sake of observational accuracy and for the correct interpretation of the effects of alternative alleles, it is important to analyze allele frequencies in relation to a given phenotype within the context of global allele frequency patterns.

The goal of our study was to identify major clinical factors associated with severe versus mild/moderate COVID-19 in patients of Uzbek ethnicity, considering two previously suggested genetic risk factors: the ACE I/D rs1799752 polymorphism and the IL28B rs12979860 polymorphism.

## Materials & methods

### Study group and study design

We performed observational retrospective study with random sampling during the patients’ hospitalization, the flowchart of the study is presented in Supplementary Fig. 1. The study population consisted of COVID-19 patients admitted to the Research Institute of Virology clinic in Tashkent, Uzbekistan, during July and August 2021 with following inclusion criteria: patients older than 18 years of self-reported Uzbek ethnicity, laboratory confirmation of COVID-19 through real-time reverse transcriptase–polymerase chain reaction (RT-PCR) and availability of a signed informed consent. SARS-CoV-2 was confirmed with RT-PCR testing of nasopharyngeal swabs, using the ROSSAmed COVID-19 RT-PCR kit (ROSSA, Uzbekistan). A total of 227 patients (12.5%) were included in the study out of 1816 patients attending the clinic, with varying degrees of COVID-19 severity. The study groups consisted of 66 patients with a mild course of the disease, 76 patients with a moderate course and 85 patients with a severe/extremely severe course. Patients were divided into mild, moderate and severe/critical groups according to the “Interim recommendations for the treatment of patients with COVID-19 coronavirus infection” of the Ministry of Health of Uzbekistan (Version 8, 2021) as described previously [9].

All individuals self-identified as belonging to the Uzbek ethnicity.

### Preparation of samples for DNA extraction

Whole blood samples from patients with COVID-19 were obtained at referral or during inpatient hospitalization. DNA extraction from peripheral blood was performed after lysis of blood erythrocytes via a DNA-Sorb-B kit (Central Research Institute of Epidemiology, Moscow, Russia). The quality of the DNA before genotyping was assessed by measuring the optical density with a UV spectrophotometer.

### Detection of ACE I/D gene polymorphism

To detect the deletion polymorphism (I/D) in the human angiotensin-converting enzyme (ACE) gene, we used the “АmpliSens ACE-I/D-EPh” kit (Central Research Institute of Epidemiology, Moscow, Russia). Positive and negative controls were included in the kit and tested at the same time as the samples. The polymerase chain reaction (PCR) products were separated and visualized on 1.7% agarose gels with ethidium bromide staining. The resulting bands of amplified DNA with a length of 422 bp correspond to the ACE insertion (I), whereas shorter 133 bp fragments correspond to the ACE deletion (D).

### Detection of IL28B gene polymorphism

To detect single nucleotide polymorphisms (SNPs) rs12979860 in the interleukin-28B (IL28B) gene via real-time PCR with hybridization-fluorescence detection, the AmpliSense^®^ Genoscreen-IL28B-FL Kit (Central Research Institute of Epidemiology, Moscow, Russia) was used. The method is based on PCR amplification with hybridization-fluorescence detection with allele-specific probes for rs12979860 and a human ß-globin probe as an endogenous internal control. Positive and negative controls were included in the kit and tested at the same time as the samples.

### Global frequency of ACE rs1799752 allele

By selecting literature that reported genotype and/or allele frequencies of the ACE I/D polymorphism, we compiled a global overview of population allele distributions to illustrate their variability across different regions. The final dataset was extracted from 95 articles available online. In most cases, we included studies with at least 100 individuals in the control group, available genotype or allele count data, and no significant deviation from Hardy–Weinberg equilibrium.

### Statistical analysis

Primer quality control of the genotyping data and genetic associations was performed with *PLINK* [10], including compliance alleles to genomic reference and test for Hardy-Weinberg equilibrium (HWE *p* = 0.08 for rs1799752 and 0.12 for rs12979860). For the three groups with different disease severity, the Student’s *t*-test was used to compare age, and Сhi-square or Fisher’s exact test was applied for categorical variables. A nonparametric univariate test was applied for associations between genetic markers and observed phenotypes, with Bonferroni correction for multiple testing. Two sets of analyses were conducted based on clinically defined groups: one comparing the mild course group with a combination of the moderate and severe/critical course groups (Severity01), and the other comparing a combination of the mild and moderate course groups with the severe/critical course group (Severity02). In logistic regression analysis, mild and moderate disease course versus severe/critical course was considered the dependent parameter tested against several clinical phenotypes and genetic markers, with age and sex included in the model with false discovery rate (FDR) for statistical correction. Statistical model testing was performed in JMP Pro 17 (JMP Statistical Discovery).

## Results

### Characteristics of the study group

This study included a cohort of 227 patients diagnosed with COVID-19. The demographic and clinical characteristics of the three patient groups are described in Table 1. The mean age of the patients was 51.1 ± 1.1 years, exhibiting a statistically significant variation across the groups, with older age manifesting in patients with severe disease. While the study did not find any statistically significant difference in disease severity based on patient sex (*p* > 0.05), female patients predominated in all the examined groups.

**Table 1:** Characteristics of patients with COVID-19

Characteristic	Mild course (*n* = 66)	Moderate course (*n* = 76)	Severe/ extremely severe course (*n* = 85)	*p*-value
Age, mean ± SD	40.4 ± 1.6	52.0 ± 1.8	61.3 ± 1.4	< 0.00001
Female, n (%)	50 (75.8%)	50 (65.8%)	47 (55.3%)	> 0.05
**Comorbidities**				
Hypertension, n (%)	8 (12.1%)	46 (60.5%)	69 (81.2%)	< 0.05
Ischemic heart disease, n (%)	0	13 (17.1%)	54 (63.5%)	< 0.05
Type 2 Diabetis, n (%)	1 (1.5%)	13 (17.1%)	33 (38.8%)	< 0.05
Obesity, n (%)	0	5 (6.6%)	13 (15.3%)	< 0.05
**Complications**				
Pneumonia, n (%)	0	69 (90.8%)	100 (100%)	< 0.05
Heart failure, n (%)	0	8 (10.5%)	42 (49.4%)	< 0.05
Acute respiratory failure, n (%)	0	3 (3.9%)	55 (64.7%)	< 0.05
Acute respiratory distress syndrome, n (%)	0	0	33 (38.8%)	< 0.05
Encephalopathy, n (%)	0	0	18 (21.2%)	< 0.05

Statistical evaluation: Student’s t-test for age and Сhi-square/Fisher’s exact test for categorical variables

The prevalence of comorbidities such as hypertension, ischaemic heart disease (IHD), and type 2 diabetes (T2D) significantly increased in the severe/extremely severe disease group (*p* < 0.05). Specifically, hypertension (*p* < 0.05), IHD (*p* < 0.05), and T2D (*p* < 0.05) were notably more prevalent in patients with severe or extremely severe disease than in those with a moderate disease course. The increase in obesity was moderate in the severe disease group.

Furthermore, patients with severe/extremely severe COVID-19 experienced a greater incidence of complications, including pneumonia (*p* < 0.05), heart failure (*p* < 0.05), acute respiratory failure (*p* < 0.05), acute respiratory distress syndrome (*p* < 0.05), and encephalopathy (*p* < 0.05), than did those with a moderate disease course (see Table 1). Importantly, however, these clinical phenotypes were used for categorizing individuals into different disease severity groups and are not independent parameters for the analyses.

The genotype and allele frequencies for two studies polymorphisms are presented in Supplementary Table 1.

### Clinical and genetic factors for predicting the severity of COVID-19

First, we performed univariate analysis of available clinical data to identify potential clinical phenotypes that correlate with the genetic markers tested in our study. The results are presented in Table 2. The severity groups were combined in two different modes with the goal of improving statistical power in the detection of possible associations: mild vs. moderate and severe (Severity01) and mild and moderate vs. severe (Severity02). Notably, no associations were found between the ACE I/D rs1799752 and IL28B rs12979860 polymorphisms and clinical phenotypes in our study population. A weak trend towards an association of hypertension with the IL28B rs12979860 polymorphism was not significant after Bonferroni correction (corrected *p* = 0.43). We therefore concluded that the clinical phenotypes that preceded infection were independent of genetic markers in our study and could be used in a multiple regression model.

**Table 2:** Univariate genetic association test in the allelic model for clinical phenotypes

Genetic marker	Severity01^1^	Severity02^2^	T2D	Heart failure	IHD	Hypertension	Obesity	Pneumonia	ARDS	Respiratory failure	Encephalpathy
ACE1 I/D	0.17	0.11	0.74	0.85	0.92	0.46	0.66	0.32	0.39	0.73	0.93
IL28B rs12979860	0.36	0.75	0.09	0.75	0.71	0.04*	0.60	0.67	0.66	0.57	0.89

1df chi-square test for allelic model, p-value

T2D - Type 2 diabetis

IHD - Ischemic heart disease

ARDS – Acute respiratory distress syndrome

^1^Corresponds to comparison of the mild course group versus a combination of the moderate and severe/critical course groups

^2^Corresponds to comparison of a combination of the mild and moderate course groups versus the severe/critical course group

*Bonferroni corrected *p* = 0.43

We found a very strong association between preinfection clinical phenotypes and disease severity in our study (Table 3), which made it difficult to identify the leading risk factor. Available clinical phenotypes, e.g., heart failure, pathological respiratory function and encephalopathy, were detected during the current study in patients with high and moderate disease severity and, to a major degree, are dependent parameters that were employed to classify individuals into groups by severity of infection. Therefore, in the main model, we included only the clinical phenotypes that preceded COVID-19 development together with the age and sex of the patients. A general evaluation of the model is presented in Table 4. We found that the overall prediction model based on selected independent parameters, including the ACE I/D polymorphism, was highly significant (Chi square 107.7, *p* < 0.0001). Concerning the specific parameters influencing the model, it became evident that previously observed IHD exerts a significant influence as a major driver with high impact. We noticed that the cumulative frequency of IHD in the groups with mild and moderate courses of COVID-19 was 9.2%, whereas in the group with severe courses, it was 63.5% (Table 1). Interestingly, the ACE I/D polymorphism was also a significant parameter in the model, with an overall FDR p value < 0.05, although these effects were weaker than those of IHD. The age of patients significantly contributed to the model, indicating a greater risk for older individuals. Surprisingly, sex did not exert a significant influence on this model (FDR p value 0.1), whereas metabolic pathology (T2D and obesity) represented only mild effects with borderline significance. The inclusion of the IL28B rs12979860 polymorphism in the model did not improve it, and this marker by itself did not contribute to the predictive value of the model.

**Table 3:** Univariate association test for clinical phenotypes and disease severity

Category	Sex	Age^1^	T2D	Heart failure	IHD	Hypertension	Obesity	Pneumonia	ARDS	Respiratory failure	Encephalpathy
**Severity01** ^2^	0.02	3.57E-12	9.17E-08	2.71E-10	4.83E-14	2.50E-17	3.13E-04	2.04E-51	6.02E-07	5.33E-12	3.13E-04
**Severity02** ^3^	0.02	2.36E-11	2.56E-10	9.57E-15	1.73E-18	8.06E-11	1.77E-03	5.35E-21	5.54E-18	1.31E-27	7.04E-10

1df chi-square test, p-value

T2D – Type 2 diabetis

IHD - Ischemic heart disease

ARDS – Acute respiratory distress syndrome

^1^Mann-Whitney test

^2^Corresponds to comparison of the mild course group versus a combination of the moderate and severe/critical course groups

^3^Corresponds to comparison of a combination of the mild and moderate course groups versus the severe/critical course group

**Table 4:** Summary of nominal logistic regression analysis for factors associated with COVID-19 severity

Model	LogLikelihood	DF	Chi Square	*P*-value, model
Difference	53.9	7	107.7	< 0.0001
**Phenotype**	**OR (95%CI)**	**FDR p-value**		
Ischemic Heart Disease	2.78 (1.80–4.28)	0.000001		
Genotype ACE (DD vs. II&ID)	1.64 (1.09–2.47)	0.0165		
Type 2 Diabetis	1.58 (1.02–2.45)	0.0367		
Obesity	2.05 (1.03–4.08)	0.0340		
Age	0.96 (0.94–0.99)	0.0069		
Sex (F)	1.37 (0.95–1.97)	0.0870		
Hypertension	1.15 (0.75–1.77)	0.5182		

The reference group: pateints with mild/moderate course of infection; combined II&ID genotype

### Allelic frequencies of ACE I/D in the Uzbek population and different populations

**Fig. 1:**
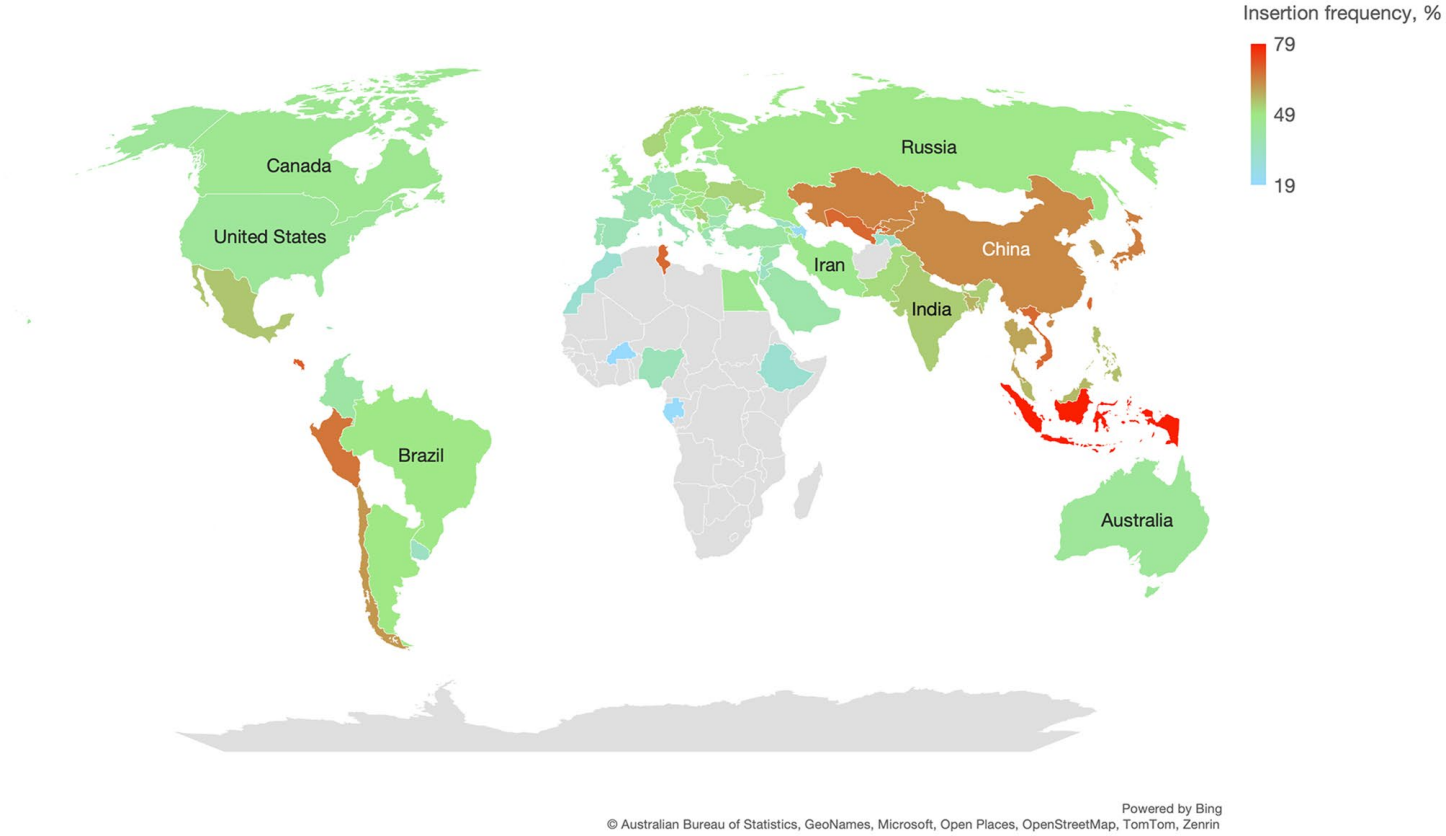
The frequency of the ACE I/D rs1799752 polymorphism worldwide

We performed critical analysis of the prevalence of ACE indels in different populations to find a possible interpretation for the discrepancies in the findings concerning the association of this genetic marker with COVID-19 severity. This is an indel of an *Alu* repetitive element in intron 16 of the ACE gene at chromosome 17q23.3. Although this polymorphism was assigned several reference sequences (rs1799752, rs4340, rs13447447, and rs4646994), it is not represented in common genetic databases because of its nature. We selected available publications from PubMed to evaluate allelic frequencies of this variation in different countries and populations. Data for 78 countries (95 studies) were extracted from the literature (Fig. 1, Supplementary Table 2). When available, we selected publications with ≥ 100 observations in the control group, with available allelic/genotyping counts and without significant deviation from Hardy‒Weinberg equilibrium (HWE). We considered such deviation as a genotyping error rather than a true distribution of genotypes due to selection or a bottleneck effect. The data for the USA, UK, Canada and Australia are presented for White Europeans. Data on allele frequency from several studies for the same countries were transformed to the weighted average value. We found that the frequency of the insertion allele (designated as I for insertion and D for deletion) varies significantly across different continents. It is a minor allele in African and European populations but has become the major allele in most East Asian populations. The data in Fig. 1 represent the insertion frequency in countries worldwide. The highest frequency of the rs1799752 insertion is clearly observed in East Asian and Southeast Asian populations, with far higher values in Indonesia and Japan, followed by China, Kazakhstan and India. In contrast, there was a clear trend toward decreasing rs1799752 insertion frequency in European populations towards western Europe. Very scarce data from the African continent do not allow conclusions to be drawn for this continent, while both Americas follow the same trend as Europe does. The insertion frequency in our study in the group with mild infection was 0.60, which is very much in line with the data from surrounding countries and reflects a high frequency of rs1799752 insertions in Asia. However, the data from some regions neighboring Uzbekistan are not available or reported with significant deviation from HWE and should be taken with caution.

Our analysis of available data for the ACE I/D rs1799752 polymorphism suggest that the spectrum of the distribution of insertion alleles in different countries may range between 0.40 and 0.80, which makes direct comparisons of the contribution of this allele to any phenotype difficult to replicate worldwide. Therefore, not directly testing for associations but including this parameter in a statistical model together with important covariates is an optimal approach for these studies. Additionally, the quality of genetic data, including HWE tests, has not been universally assessed in available publications, which may cause confusion in the interpretation of results.

## Discussion

Our findings indicate that ischaemic heart disease (IHD), alongside the ACE I/D polymorphism and patient age, are significant factors contributing to the severity of COVID-19 infection in the Uzbek population. The data underscore the critical interplay between these variables in influencing disease outcomes, suggesting that both genetic and clinical characteristics must be considered when assessing risk and managing treatment strategies for COVID-19 in this population.

By April 2024, marking the end of official reporting, over 253,600 cases of COVID-19 have been documented in Uzbekistan [11]. Currently, COVID-19 infection does not have a high mortality rate worldwide because of large-scale population vaccination programs. However, cases of severe disease and death persist among vulnerable populations, especially among unvaccinated individuals and in persons with comorbidities. Although the majority of infected patients with COVID-19 develop pneumonia, this disease represents a multifaceted pathophysiological condition, and affected organs include not only the lungs but also the heart and other organs. There have been numerous attempts to identify major factors that affect disease development, especially its most severe form. During the first year of the pandemic, Tao Zhang and colleagues conducted a meta-analysis to identify major clinical characteristics that differ between severe and nonsevere COVID-19 patients, with sixteen studies including 1,172 patients with severe outcomes and 2,803 patients with nonsevere outcomes. Various comorbidities, including hypertension, cardiovascular diseases, COPD, and diabetes, have been identified as risk factors for a more severe course of the disease and increased mortality [12]. Many studies have demonstrated that elderly individuals, a vulnerable population with chronic conditions such as cardiovascular disease, pulmonary disease, and diabetes, are at increased risk of developing severe COVID-19, and overall, preceding chronic diseases increase the risk of severe COVID-19 [13–19]. The results of our study demonstrated that a history of IHD, T2D, and obesity, along with older age and the presence of the DD genotype of the ACE I/D polymorphism, collectively contribute to a more severe course of COVID-19.

With pandemic expansion, it has become even more evident that host factors may play a crucial role in determining the clinical presentation and outcomes of COVID-19 infection. Therefore, the COVID-19 pandemic has sparked a concentrated interest in genetic polymorphisms correlated with susceptibility to and severity of the disease. Genome-wide association studies (GWASs) have revealed several variations annotated to multiple genes. Among those genes, at least two, ACE2 and SLC6A20, are involved in the renin‒angiotensin pathway [5]. Owing to the role of the ACE2 receptor in the entry mechanism of coronavirus through angiotensin-converting enzyme 2, the SARS-CoV-2 cell-surface receptor, it is logical to assume that other members of the renin‒angiotensin system (RAS) may also play a role in COVID-19-related phenotypes. Multiple studies have explored the significance of important members of the RAS in patients with COVID-19, and a significant fraction of these studies considered genetic polymorphisms in the ACE gene.

The ACE gene, located on chromosome 17q23.3, spans a length of 21.32 kb and includes 26 exons. The most studied genetic variation within the ACE gene is the insertion/deletion (I/D) polymorphism within intron 16 (rs1799752, aka rs4340, rs13447447, rs4646994) [20–22]. Consequently, in the human population, the I/D polymorphism is characterized by three genotypes: II, ID, and DD. We conducted a systematic analysis of allelic frequencies for this polymorphism using global population data available in the literature. Notably, we observed considerable variability in genotyping quality, often indicated by deviations from Hardy‒Weinberg equilibrium (HWE). Caution is warranted when interpreting data from studies with such deviations. In our summary (Supplementary Table 2), we cite only six studies with significant deviation from HWE, and this is limited to cases where no alternative data were available for the respective countries. Additionally, in seven studies, we found no statements regarding HWE and no genotyping counts to test for HWE. All referenced studies were chosen on the basis of a substantial number of observations in the healthy control group. However, in 15 cases, the sample size was less than 100 because of the absence of alternative data from those regions.

Research results concerning the association of ACE I/D polymorphisms with the severity of COVID-19 at an early stage have revealed the importance of certain comorbidities in this association. A study by Gomes et al. demonstrated that the ACE I/D polymorphism was associated with the risk of developing severe COVID-19, depending on hypertension status [8]. The overall frequency of deletions may be positively correlated with mortality from COVID-19 [23]. In contrast, in a study by Faridzadeh A et al., although a correlation of this polymorphism with chronic diseases and with susceptibility to COVID-19 was not found, the frequency of the ACE DD genotype inversely correlated with severe outcomes in COVID-19 patients [24]. Another study from the same population, however, confirmed the ACE1 DD genotype as a risk factor for severe COVID-19 infection [25]. Several other studies have reported associations between the ACE D allele or DD genotype and the risk of developing severe COVID-19 and worsening adverse outcomes in different countries [26, 27].

To investigate whether the ACE1 I/D polymorphism is associated with the severity of COVID-19, a meta-analysis was conducted, including 11 studies with 692 individuals with severe COVID-19 and 1433 individuals with mild manifestations of the disease. However, this study ignored significant differences in the allelic frequency of the ACE1 I/D polymorphism in different populations. This issue, together with deviation from HWE in some studies, resulted in a very high heterogeneity index (I^2^ = 87–92%) and difficulties in interpreting the results [28]. Another meta-analysis of the association of this polymorphism with the severity of COVID-19 revealed that 4 studies and 718 participants were less affected by population heterogeneity, resulting in a significant association between the DD genotype and the severity of COVID-19 [29].

Interestingly, the ACE1 I/D polymorphism was previously associated with acute respiratory distress syndrome [30].

Ethnic and geographic differences in ACE1 gene polymorphisms vary widely. Analysis of epidemiological data from 26 European countries at the beginning of the pandemic revealed a positive correlation between the frequency of the D allele in the population (indicated range between 0.51 and 0.66) and mortality from COVID-19 in the same population [23]. This was not confirmed in another similar analysis of data from 18 European countries [31]. However, while it remains an important cofounder, the ethnic diversity within the country was not taken into consideration in these studies. On the other hand, the European population has a higher frequency of the ACE DD genotype [32] and a higher prevalence and mortality from COVID-19 than the Asian population does [33]. The Uzbek population is genetically heterogeneous, shaped by both Western and Eastern Eurasian ancestry, which distinguishes it from more homogeneous European or East Asian cohorts. Our data suggest that the profile of ACE1 I/D polymorphisms in this population is representative of most East Asian populations. This heterogeneity may influence both genetic susceptibility and clinical presentation, underscoring the importance of population-specific analyses in our study.

The relationship between ACE1 I/D polymorphisms and disease severity differs among populations around the globe [34, 35]. The distribution of the D allele is characterized by the highest frequency in Africa and Arab regions; moderate frequency in Europe, Australia, and America; and the lowest frequency in East Asia [32, 36].

We did not find studies on the ACE1 polymorphism in COVID-19 among the population of Uzbekistan. However, the frequency of this polymorphism has been studied in Uzbek patients with cardiovascular diseases. In the study by Kurbanov R et al., which focused on individuals of Uzbek nationality suffering from dilated cardiomyopathy, the prevalence of the ID heterozygous genotype (44.1%) and the I allele (54.4%) was shown, whereas the II genotype (56.7%) and I allele (65.8%) were more commonly detected in healthy individuals [37].

The results of our research confirm the significance of cardiovascular clinical phenotypes that precede severe COVID-19 infection, and the data suggest the contribution of ACE genetics to the development of severe COVID-19 infection in this population.

A plausible mechanism linking the ACE I/D polymorphism to COVID-19 severity is via modulation of the renin–angiotensin system (RAS). The I/D polymorphism influences circulating ACE activity and the balance between angiotensin II and other types of angiotensin [22]. Increased ACE activity (classically associated with the D allele) promotes higher angiotensin II levels, which can drive vasoconstriction, endothelial dysfunction, oxidative stress, inflammation and pro-thrombotic states [22]. All these factors predispose to cardiovascular disease (including IHD) and may worsen COVID-19 outcomes. In addition, dysregulated RAS signaling is implicated in insulin resistance, adipose inflammation and other metabolic perturbations (T2D, obesity), providing a plausible pathway by which ACE genotype could indirectly amplify COVID-19 severity via comorbid cardiometabolic disease [38]. These mechanisms are consistent with our observation that IHD, T2D, obesity and older age are the major clinical drivers of severity in our cohort. However, as causal inference is limited in an observational study, targeted biochemical and functional studies are required to confirm these pathways, and the proposed mechanistic explanations should be interpreted with caution.

Considering the central role of host genes in shaping the immune response, several genetic variations within immune system-related genes, including IFNL3 (IL28B) polymorphisms, have been explored for their associations with COVID-19 severity. Interferons play crucial roles in the outcome of COVID-19 infection, and variations in the IFNA10 and INFAR2 genes have been detected in association with critical cases of COVID-19 [5]. Despite multiple attempts to address the role of IFNL3 (IL28B) polymorphisms in COVID-19, neither GWAS nor meta-analyses of published data have shown such associations [5, 29, 39, 40]. Our study also revealed no association of IFNL3 (IL28B) polymorphisms with COVID-19 severity in the Uzbek population.

Our findings suggest that certain comorbidities, particularly IHD, but also T2D and obesity, together with older age, are major contributors to COVID-19 severity in the Uzbek population, while sex appears to play a limited role. Clinically, this underscores the importance of early identification and careful monitoring of patients with these risk factors. While our study does not directly inform treatment strategies, it supports the value of incorporating comorbidities and age into risk stratification and preventive approaches, which may guide patient management and resource allocation during outbreaks.

Our extensive examination of the allelic frequency for the ACE I/D polymorphism in global population shows a broad range in the prevalence of particular alleles. Thus, it is crucial to consider the directionality of association and the size of the research population based on local allelic frequency when conducting association studies, and particularly in replication studies. In ethnically diverse populations, the impact of the main allele and the statistical power of the study may differ substantially. As evidenced by numerous publications with suboptimal genotyping techniques and no control for HWE, the disregard for standard metrics for genotyping quality also results in poorly interpretable data.

The major limitations of our study include the relatively small number of observations and the potential bias due to the selective nature of patients admitted to the Research Institute of Virology. Another limitation is the very limited number of genetic markers analysed, which cannot adequately capture the full genetic landscape influencing COVID-19 severity. These genetic markers, although previosly suggested, did not appear in the largest GWAS study of COVID-19 severity [41–43]. This discordance could be attributed to several factors. The most apparent reasons include the absence of an actual association, the non-conformity of the variation type (e.g. ACE I/D is not directly detected in SNP-based GWAS), a substantial ancestry bias (> 80% Europeans, with minimal contribution from Asians), and the univariate analysis design which overlooks significant clinical phenotypes, such as IHD. Due to time and budget restriction, our study lacks validation cohort. We consider our findings exploratory, aiming to investigate the combinatorial effects of significant clinical risk factors alongside genetic factors. Expending the model to include additional genetic markers, such as those highlighted in the referenced publication, would be a valuable step forward. Furthermore, the strength of the impacts from the statistical model should be interpreted very carefully because the clinical predisease phenotypes, such as IHD, T2D, and hypertension, are known to be strongly correlated with age. Finally, the study identifies associations but does not establish causality between genetic polymorphisms and COVID-19 severity, which may result from a complex interplay between genetic background and environmental factors.

It is important to note, that our Uzbek study population was small and extremely selective. Therefore, our conclusions cannot be applied directly to populations of other nationalities or to the broader community. More extensive research is required, encompassing a wider range of ethnic groups. Our study supported earlier recommendations [44, 45] that close monitoring for early indicators of COVID-19 and its possible progression is essential for patients with IHD. Optimizing treatment adherence, modifying lifestyle factors, and guaranteeing priority COVID-19 vaccination are important goals. To reduce health risks, avoid problems, and keep the patient’s condition from getting worse, special attention should be paid to tailoring the COVID-19 treatment strategy while considering the unique features of IHD. The strength is the combination of genetic and clinical data in a single model, which is a more holistic approach. Future directions include replicating this study in different populations, followed by meta-analyses that take into account the allelic frequencies of the ACE I/D polymorphism.

## Conclusion

In summary, within a cohort of patients from the Uzbek population, we reaffirmed the significance of IHD, age and metabolic disorders preceding severe COVID-19 infection. Our findings indicate a potential contribution from ACE genetics to the development of severe COVID-19 infection in this population. There are further studies required to be carried out on the potential genetic contribution of ACE I/D polymorphism to the global population.

## Electronic Supplementary Material

Below is the link to the electronic supplementary material.

Supplementary Material 1: Supplementary Figure 1. Study Design and Genetic Analyses Flowchart

Supplementary Material 2: Supplementary Table 1. Genotype and allele frequencies

Supplementary Material 3: Supplementary Table 2. Allelic and genotype frequencies of ACE I/D polymorphisms in the global population

## Data Availability

Data is provided within the manuscript or supplementary information files.
