# Supplementary material for: Ischaemic heart disease is the factor associated with severe COVID-19 in the urban population of Uzbekistan: a single‑center retrospective study
Source: BMC Infect Dis. 2026 Feb 12;26:581. doi: 10.1186/s12879-026-12798-6 (PMC12998015; doi:10.1186/s12879-026-12798-6)
Supplement: Supplementary file 1 — Supplementary Material 1: Supplementary Figure 1. Study Design and Genetic Analyses Flowchart [file 12879_2026_12798_MOESM1_ESM.pdf]

Supplementary Figure 1. Study Design and Genetic Analyses Flowchart

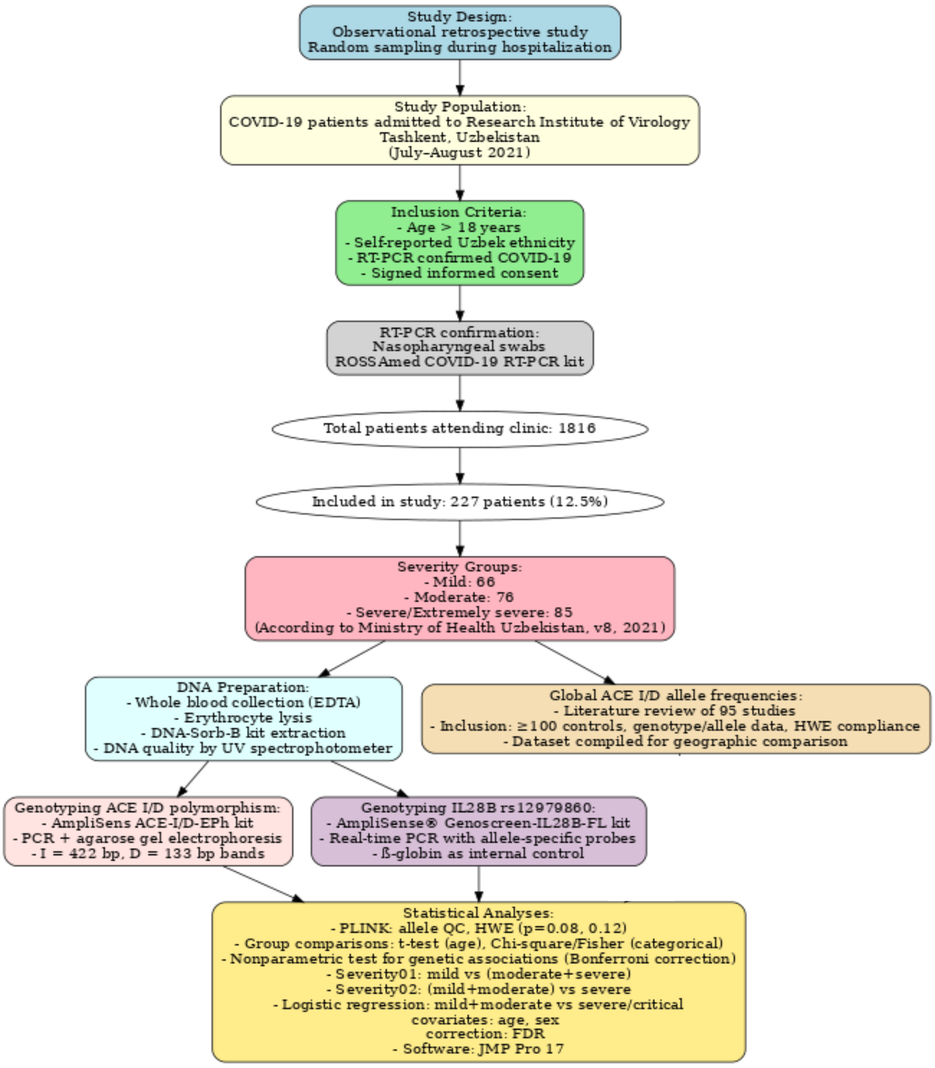

Flowchart of study design and analyses. COVID-19 patients in Tashkent, Uzbekistan, were stratified by disease severity (mild, moderate, severe/critical). Peripheral blood DNA was extracted and genotyped for ACE I/D and IL28B rs12979860 polymorphisms. Genetic associations with disease severity were assessed for group comparisons, and logistic regression adjusted for age and sex.
