# Supplementary material for: Ischaemic heart disease is the factor associated with severe COVID-19 in the urban population of Uzbekistan: a single‑center retrospective study
Source: BMC Infect Dis. 2026 Feb 12;26:581. doi: 10.1186/s12879-026-12798-6 (PMC12998015; doi:10.1186/s12879-026-12798-6)
Supplement: Supplementary file 2 — Supplementary Material 2: Supplementary Table 1. Genotype and allele frequencies [file 12879_2026_12798_MOESM2_ESM.docx]

Supplementary Table 1. Genotype and allele frequencies

|  | Number of observations | |  |  | Frequency of genotype/allele | |  |
| --- | --- | --- | --- | --- | --- | --- | --- |
|  | Mild course | Moderate course | Severe/ extremely severe course |  | Mild course | Moderate course | Severe/ extremely severe course |
| ACE I/D rs1799752 | n=66 | n=76 | n=85 |  |  |  |  |
| DD | 12 | 16 | 25 |  | 0.182 | 0.211 | 0.294 |
| DI | 29 | 35 | 35 |  | 0.439 | 0.461 | 0.412 |
| II | 25 | 25 | 25 |  | 0.379 | 0.329 | 0.294 |
| Allele D | 53 | 67 | 85 |  | 0.402 | 0.441 | 0.500 |
| Allele I | 79 | 85 | 85 |  | 0.598 | 0.559 | 0.500 |
|  |  |  |  |  |  |  |  |
| IL28B rs12979860 | n=60 | n=70 | n=68 |  |  |  |  |
| CC | 35 | 48 | 41 |  | 0.583 | 0.686 | 0.603 |
| CT | 23 | 21 | 26 |  | 0.383 | 0.300 | 0.382 |
| TT | 2 | 1 | 1 |  | 0.033 | 0.014 | 0.015 |
| Allele C | 93 | 117 | 108 |  | 0.775 | 0.836 | 0.794 |
| Allele T | 27 | 23 | 28 |  | 0.225 | 0.164 | 0.206 |
