# Supplementary material for: Ischaemic heart disease is the factor associated with severe COVID-19 in the urban population of Uzbekistan: a single‑center retrospective study
Source: BMC Infect Dis. 2026 Feb 12;26:581. doi: 10.1186/s12879-026-12798-6 (PMC12998015; doi:10.1186/s12879-026-12798-6)
Supplement: Supplementary file 3 — Supplementary Material 3: Supplementary Table 2. Allelic and genotype frequencies of ACE I/D polymorphisms in the global population [file 12879_2026_12798_MOESM3_ESM.docx]

Supplementary Table 2. Allelic and genotype frequencies of ACE I/D polymorphism in the global population.

| Country | Reference | First author | Year | Number of controls | II | ID | DD | II, % | ID, % | DD, % | Insertion frequency, % | HWE^1^ |
| --- | --- | --- | --- | --- | --- | --- | --- | --- | --- | --- | --- | --- |
| Albania | [1] | Comas et al. | 2004 | 60 | NA | NA | NA | NA | NA | NA | 47 | ok |
| Argentina | [2] | Jiménez et al. | 2007 | 75 | 16 | 42 | 17 | 21 | 56 | 23 | 49 | ok |
| Armenia | [3] | Romualdi et al. | 2002 | 43 | NA | NA | NA | NA | NA | NA | 48 | NA |
| Australia | [4] | Lea et al. | 2005 | 244 | 46 | 122 | 76 | 19 | 50 | 31 | 44 | ok |
| Austria | [5] | Renner et al. | 2002 | 522 | 124 | 252 | 146 | 24 | 48 | 28 | 46 | ok |
| Azerbaijan | [3] | Romualdi et al. | 2002 | 37 | NA | NA | NA | NA | NA | NA | 22 | NA |
| Bangladesh | [6] | Hosen et al. | 2021 | 120 | 37 | 62 | 21 | 31 | 51.5 | 17.5 | 57 | ok |
| Belgium | [7] | Li et al. | 2007 | 1287 | 306 | 665 | 316 | 23 | 52 | 25 | 50 | ok |
| Bosnia and Herzegovina | [8] | Cenanovic et al. | 2021 | 242 | NA | NA | NA | NA | NA | NA | 43 | NA |
| Brazil | [9] | Kimura et al. | 2012 | 206 | 54 | 92 | 60 | 26 | 45 | 29 | 49 | ok |
| Brazil | [10] | Schuch et al. | 2014 | 205 | 54 | 101 | 50 | 27 | 49 | 24 | 51 | ok |
| Brazil total |  |  |  | 411 |  |  |  |  |  |  | 50 |  |
| Bulgaria | [11] | Kostadinova et al. | 2017 | 109 | 16 | 53 | 40 | 15 | 49 | 37 | 39 | ok |
| Burkina Faso | [12] | Tchelougou et al. | 2015 | 202 | 10 | 57 | 135 | 5 | 28 | 67 | 19 | ok |
| Canada | [13] | Sun et al. | 2018 | 1652 | 351 | 829 | 472 | 21 | 50 | 29 | 46 | ok |
| Chile | [14] | Sanhueza et al. | 2016 | 192 | 75 | 86 | 31 | 39 | 45 | 16 | 61 | ok |
| China | [15] | Young et al. | 1998 | 183 | 71 | 88 | 24 | 39 | 48 | 13 | 63 | ok |
| China | [16] | Ohishi et al. | 1994 | 111 | 40 | 50 | 21 | 36 | 45 | 19 | 59 | ok |
| China | [17] | Thomas et al. | 2001 | 119 | 39 | 61 | 19 | 33 | 51 | 16 | 58 | ok |
| China | [18] | Fan et al. | 2007 | 951 | 311 | 427 | 183 | 34 | 46 | 20 | 57 | ok |
| China total |  |  |  | 1364 |  |  |  |  |  |  | 58 |  |
| Colombia | [19] | Bautista et al. | 2008 | 191 | 38 | 116 | 77 | 17 | 50 | 33 | 42 | ok |
| Costa Rica | [20] | Herrmann et al. | 2004 | 728 | 350 | 313 | 65 | 48 | 43 | 9 | 70 | ok |
| Croatia | [21] | Nadalin et al. | 2017 | 521 | 95 | 276 | 150 | 18 | 53 | 29 | 45 | ok |
| Cyprus | [3] | Romualdi et al. | 2002 | 49 | NA | NA | NA | NA | NA | NA | 33 | NA |
| Czech | [22] | Bartakova et al. | 2022 | 230 | 31 | 74 | 38 | 22 | 52 | 27 | 48 | ok |
| Denmark | [23] | Bladbjerg et al. | 1999 | 199 | 46 | 102 | 51 | 23 | 51 | 26 | 49 | ok |
| Egypt | [24] | Taha et al. | 2024 | 100 | 21 | 50 | 29 | *21* | 50 | 29 | 46 | ok |
| Egypt | [25] | Boraey et al. | 2024 | 460 | 106 | 225 | 129 | 23 | 49 | 28 | 48 | ok |
| Egypt | [26] | AbdRaboh et al. | 2013 | 93 | 16 | 52 | 25 | 17 | 56 | 27 | 45 | ok |
| Egypt total |  |  |  | 653 |  |  |  |  |  |  | 47 |  |
| Estonia | [27] | Mäestu et al. | 2013 | 261 | 67 | 114 | 80 | 26 | 44 | 30 | 48 | 0.05 |
| Ethiopia | [28] | Ash et al. | 2011 | 315 | 24 | 144 | 147 | 8 | 46 | 47 | 31 | ok |
| Finland | [29] | Lahtela et al. | 2017 | 149 | 37 | 71 | 41 | 25 | 48 | 27 | 49 | ok |
| France | [30] | Hadjadj et al. | 2008 | 1277 | 192 | 612 | 473 | 15 | 48 | 37 | 39 | ok |
| Gabon | [31] | Ndong et al. | 2017 | 88 | 3 | 29 | 56 | 3 | 33 | 34 | 20 | ok |
| Georgia | [3] | Romualdi et al. | 2002 | 65 | NA | NA | NA | NA | NA | NA | 35 | NA |
| Germany | [32] | Filler et al. | 2001 | 100 | 15 | 47 | 38 | 15 | 47 | 38 | 39 | ok |
| Germany | [33] | Ebert et al. | 2005 | 145 | 33 | 72 | 40 | 23 | 50 | 27 | 48 | ok |
| Germany | [34] | Mohlendick et al. | 2021 | 200 | 50 | 95 | 55 | 25 | 47.5 | 27.5 | 49 | ok |
| Germany total |  |  |  | 445 |  |  |  |  |  |  | 46 |  |
| Greece | [35] | Eleni et al. | 2008 | 352 | 46 | 178 | 128 | 13 | 51 | 36 | 38 | ok |
| Greece | [36] | Sgourou et al. | 2012 | 171 | 26 | 88 | 57 | 15 | 52 | 33 | 41 | ok |
| Greece | [37] | Kolovou et al. | 2013 | 100 | 18 | 46 | 36 | 18 | 46 | 36 | 41 | ok |
| Greece total |  |  |  | 623 |  |  |  |  |  |  | 40 |  |
| Hungary | [38] | Barkai et al. | 2005 | 120 | 33 | 57 | 34 | 27 | 46 | 27 | 50 | ok |
| India | [39] | Gupta et al. | 2009 | 110 | 33 | 50 | 27 | 30 | 45.5 | 24.5 | 53 | ok |
| India | [40] | Jhawat et al. | 2019 | 270 | 60 | 140 | 70 | 22 | 52 | 26 | 48 | ok |
| India | [41] | Patel et al. | 2022 | 292 | 61 | 159 | 72 | 21 | 54 | 25 | 48 | ok |
| India total |  |  |  | 672 |  |  |  |  |  |  | 49 |  |
| Indonesia | [42] | Bawazier et al. | 2010 | 108 | 66 | 38 | 4 | 61 | 35 | 4 | 79 | ok |
| Iran | [43] | Abbaszadeh et al. | 2022 | 194 | 41 | 101 | 52 | 21 | 52 | 27 | 47 | ok |
| Italy | [44] | Panza et al. | 2002 | 268 | 32 | 138 | 98 | 12 | 52 | 37 | 38 | ok |
| Italy | [45] | Di Pasquale et al. | 2005 | 684 | 124 | 335 | 225 | 18 | 49 | 33 | 43 | ok |
| Italy total |  |  |  | 952 |  |  |  |  |  |  | 42 |  |
| Izrael | [46] | Amir et al. | 2007 | 247 | 26 | 115 | 106 | 10 | 46 | 43 | 34 | ok |
| Jamaica | [47] | Kramer et al. | 2005 | 428 | NA | NA | NA | NA | NA | NA | 38 | ok |
| Japan | [48] | Mannami et al. | 2001 | 3657 | 1540 | 1640 | 477 | 42 | 45 | 13 | 65 | ok |
| Japan | [49] | Wakai et al. | 2011 | 4509 | 1854 | 2021 | 634 | 41 | 45 | 14 | 64 | ok |
| Japan total |  |  |  | 8166 |  |  |  |  |  |  | 64 |  |
| Jordan | [50] | AL-Eitan et al. | 2023 | 525 | 56 | 248 | 221 | 11 | 47 | 42 | 34 | ok |
| Kazakhstan | [51] | Svyatova et al. | 2023 | 1801 | 753 | 798 | 250 | 42 | 44 | 14 | 64 | ok |
| Korea, South | [52] | Um et al. | 2003 | 613 | 225 | 295 | 93 | 37 | 48 | 15 | 61 | ok |
| Korea, South | [53] | Kwon | 2020 | 353 | 124 | 177 | 52 | 35 | 50 | 15 | 60 | ok |
| Korea, South total |  |  |  | 966 |  |  |  |  |  |  | 61 |  |
| Kyrgyzstan | [54] | Khitrinskaya et al. | 2003 | 104 | 41 | 49 | 14 | 39 | 47 | 14 | 63 | ok |
| Latvia | [55] | Paulauskas et al. | 2009 | 116 | 27 | 47 | 42 | 23 | 41 | 36 | 43 | ok |
| Lebanon | [56] | Saab et al. | 2007 | 570 | 42 | 219 | 309 | 7 | 40 | 53 | 27 | ok |
| Lebanon | [57] | Saad et al. | 2023 | 155 | 12 | 72 | 71 | 8 | 46 | 46 | 31 | ok |
| Lebanon total |  |  |  | 725 |  |  |  |  |  |  | 28 |  |
| Malaysia | [58] | Wei et al. | 2015 | 297 | 90 | 154 | 53 | 30 | 52 | 18 | 56 | ok |
| Mexico | [59] | Thameem et al. | 2008 | 670 | 181 | 348 | 141 | 27 | 52 | 21 | 54 | ok |
| Moldova | [60] | Capros et al. | 2013 | 290 | 96 | 162 | 32 | 33.11 | 55.86 | 11.03 | 39 | 0.004 |
| Morocco | [61] | Comas et al. | 2000 | 300 | NA | NA | NA | NA | NA | NA | 30 | NA |
| Netherlands | [62] | van der Knaap et al. | 2008 | 6670 | 1473 | 3335 | 1862 | 22 | 50 | 28 | 47 | ok |
| Nigeria | [47] | Kramer et al. | 2005 | 1059 | NA | NA | NA | NA | NA | NA | 36 | ok |
| Nigeria | [63] | Kooffreh et al. | 2014 | 612 | 74 | 303 | 235 | 12 | 50 | 38 | 37 | ok |
| Nigeria total |  |  |  | 1671 |  |  |  |  |  |  | 36 |  |
| North Macedonia | [1] | Comas et al. | 2004 | 55 | NA | NA | NA | NA | NA | NA | 48 | ok |
| Norway | [64] | Tronvik et al. | 2008 | 403 | 107 | 204 | 92 | 26 | 51 | 23 | 52 | ok |
| Pakistan | [65] | Mansoor et al. | 2012 | 276 | 59 | 161 | 56 | 22 | 58 | 20 | 51 | 0.01 |
| Peru | [66] | Oscanoa et al. | 2020 | 104 | 46 | 45 | 13 | 44 | 44 | 12 | 66 | ok |
| Philippines | [67] | Grandinetti et al. | 2006 | 95 | 28 | 49 | 18 | 29 | 52 | 19 | 55 | ok |
| Poland | [68] | Goracy et al. | 2022 | 152 | 36 | 81 | 35 | 24 | 53 | 23 | 50 | ok |
| Portugal | [69] | Sousa et al. | 2018 | 852 | 128 | 389 | 335 | 15 | 46 | 39 | 38 | ok |
| Romania | [70] | Toma et al. | 2009 | 150 | 30 | 73 | 47 | 20 | 49 | 31 | 45 | ok |
| Romania | [71] | Procopciuc et al. | 2018 | 130 | 49 | 51 | 30 | 38 | 39 | 23 | 57 | 0.03 |
| Romania total |  |  |  | 280 |  |  |  |  |  |  | 50 |  |
| Russia | [72] | Gineviciene et al. | 2016 | 947 | 235 | 444 | 268 | 25 | 47 | 28 | 48 | ok |
| Russia | [73] | Bondarenco et al. | 2016 | 199 | 52 | 98 | 49 | 26 | 49 | 25 | 51 | ok |
| Russia total |  |  |  | 1146 |  |  |  |  |  |  | 49 |  |
| Saudi Arabia | [74] | Al-Harbi et al. | 2015 | 145 | 19 | 62 | 44 | 15 | 50 | 35 | 40 | ok |
| Serbia | [75] | Stankovic et al. | 2023 | 100 | 23 | 48 | 29 | 23 | 48 | 29 | 53 | ok |
| Slovakia | [76] | Siváková et al. | 2009 | 209 | 52 | 95 | 62 | 25 | 45 | 30 | 48 | ok |
| Slovenia | [21] | Nadalin et al. | 2017 | 521 | 95 | 276 | 150 | 18 | 53 | 29 | 45 | ok |
| Spain | [77] | Alvarez et al. | 1999 | 400 | 60 | 176 | 164 | 15 | 44 | 41 | 37 | ok |
| Spain | [78] | Romero-Blanco et al. | 2020 | 282 | 46 | 131 | 105 | 16 | 47 | 37 | 40 | ok |
| Spain total |  |  |  | 682 |  |  |  |  |  |  | 38 |  |
| Sweden | [79] | Bengtsson et al. | 1999 | 1149 | 264 | 598 | 287 | 23 | 52 | 25 | 49 | ok |
| Switzerland | [80] | Walder et al. | 1998 | 199 | 49 | 92 | 58 | 25 | 46 | 29 | 48 | ok |
| Syria | [3] | Romualdi et al. | 2002 | 70 | NA | NA | NA | NA | NA | NA | 40 | NA |
| Taiwan | [81] | Lee et al. | 2002 | 750 | 350 | 322 | 78 | 47 | 43 | 10 | 68 | ok |
| Tajikistan | [54] | Khitrinskaya et al. | 2003 | 41 | 3 | 21 | 17 | 7 | 51 | 42 | 33 | ok |
| Thailand | [82] | Chutinet et al. | 2012 | 167 | 64 | 68 | 35 | 38 | 41 | 21 | 59 | 0.04 |
| Tunisia | [83] | Ezzidi et al. | 2009 | 473 | 356 | 311 | 81 | 48 | 41 | 11 | 68 | ok |
| Tunisia | [84] | Mehri et al | 2010 | 238 | 81 | 106 | 51 | 34 | 45 | 21 | 56 | ok |
| Tunisia total |  |  |  | 711 |  |  |  |  |  |  | 64 |  |
| Turkey | [85] | Bedir et al. | 1999 | 143 | 19 | 82 | 42 | 13 | 57 | 30 | 42 | ok |
| Turkey | [86] | Serdaroglu et al. | 2005 | 287 | 64 | 124 | 99 | 22 | 43 | 35 | 44 | 0.04 |
| Turkey total |  |  |  | 430 |  |  |  |  |  |  | 43 |  |
| Ukraine | [87] | Drozdovska et al. | 2013 | 283 | 71 | 150 | 62 | 25 | 53 | 22 | 52 | ok |
| United Kingdom | [88] | Kehoe et al. | 1999 | 386 | 89 | 180 | 117 | 23 | 47 | 30 | 46 | ok |
| United Kingdom | [89] | Keavney et al. | 2000 | 5934 | 1317 | 2980 | 1637 | 22 | 50 | 28 | 47 | ok |
| United Kingdom | [90] | Steeds et al. | 2001 | 507 | 112 | 237 | 158 | 22 | 47 | 31 | 45 | ok |
| United Kingdom total |  |  |  | 6827 |  |  |  |  |  |  | 47 |  |
| Uruguay | [91] | Hidalgo et al. | 2014 | 67 | NA | NA | NA | NA | NA | NA | 34 | ok |
| USA | [92] | Miners et al. | 2009 | 135 | 30 | 59 | 46 | 22 | 44 | 34 | 44 | ok |
| Uzbekistan | [93] | Bakhtiyarova et al. | 2014 | 60 | 34 | 14 | 12 | 57 | 23 | 20 | 68 | 0.0006 |
| Uzbekistan | [94] | Kan et al. | 2012 | 45 | 21 | 19 | 5 | 47 | 42 | 11 | 68 | ok |
| Uzbekistan | [54] | Khitrinskaya et al. | 2003 | 46 | 18 | 19 | 9 | 39 | 41 | 20 | 60 | ok |
| Uzbekistan total |  |  |  | 151 |  |  |  |  |  |  | 66 |  |
| Vietnam | [95] | Itoyama et al. | 2003 | 153 | 69 | 69 | 15 | 45 | 45 | 10 | 68 | ok |

I – insertion; D – deletion; NA – not available in referred article.

^1^HWE – Hardy-Weinberg equilibrium test; ok - >0.05.

1. Comas, D., et al., *Alu insertion polymorphisms in the Balkans and the origins of the Aromuns.* Ann Hum Genet, 2004. **68**(Pt 2): p. 120-7.

2. Jimenez, P.M., et al., *Association of ACE genotype and predominantly diastolic hypertension: a preliminary study.* J Renin Angiotensin Aldosterone Syst, 2007. **8**(1): p. 42-4.

3. Romualdi, C., et al., *Patterns of human diversity, within and among continents, inferred from biallelic DNA polymorphisms.* Genome Res, 2002. **12**(4): p. 602-12.

4. Lea, R.A., et al., *Genetic variants of angiotensin converting enzyme and methylenetetrahydrofolate reductase may act in combination to increase migraine susceptibility.* Brain Res Mol Brain Res, 2005. **136**(1-2): p. 112-7.

5. Renner, W., et al., *The angiotensin-converting-enzyme insertion/deletion polymorphism is not a risk factor for peripheral arterial disease.* Atherosclerosis, 2002. **165**(1): p. 175-8.

6. Hosen, M.B., et al., *Assessment of angiotensin converting enzyme gene polymorphism in preeclampsia mothers of Bangladesh.* J Obstet Gynaecol, 2021. **41**(7): p. 1032-1035.

7. Li, Y., et al., *Angiotensin-converting enzyme I/D and alpha-adducin Gly460Trp polymorphisms: from angiotensin-converting enzyme activity to cardiovascular outcome.* Hypertension, 2007. **49**(6): p. 1291-7.

8. Cenanovic, M., et al., *Distribution of the ACE1 D Allele in the Bosnian-Herzegovinian Population and its Possible Role in the Regional Epidemiological Picture of COVID-19.* Genet Test Mol Biomarkers, 2021. **25**(1): p. 55-58.

9. Kimura, L., et al., *Multilocus family-based association analysis of seven candidate polymorphisms with essential hypertension in an african-derived semi-isolated brazilian population.* Int J Hypertens, 2012. **2012**: p. 859219.

10. Schuch, J.B., et al., *ACE polymorphism and use of ACE inhibitors: effects on memory performance.* Age (Dordr), 2014. **36**(3): p. 9646.

11. Kostadinova, E.S., L.D. Miteva, and S.A. Stanilova, *ACE serum level and I/D gene polymorphism in children with obstructive uropathies and other congenital anomalies of the kidney and urinary tract.* Nephrology (Carlton), 2017. **22**(8): p. 609-616.

12. Tchelougou, D., et al., *Renin-Angiotensin System Genes Polymorphisms and Essential Hypertension in Burkina Faso, West Africa.* Int J Hypertens, 2015. **2015**: p. 979631.

13. Sun, C., et al., *Childhood adiposity, adult adiposity, and the ACE gene insertion/deletion polymorphism: evidence of gene-environment interaction effects on adult blood pressure and hypertension status in adulthood.* J Hypertens, 2018. **36**(11): p. 2168-2176.

14. Sanhueza, J.A., et al., *Association of Anxiety-Related Polymorphisms with Sports Performance in Chilean Long Distance Triathletes: A Pilot Study.* J Sports Sci Med, 2016. **15**(4): p. 554-561.

15. Young, R.P., et al., *Angiotensinogen T235 and ACE insertion/deletion polymorphisms associated with albuminuria in Chinese type 2 diabetic patients.* Diabetes Care, 1998. **21**(3): p. 431-7.

16. Ohishi, M., H. Rakugi, and T. Ogihara, *Association between a deletion polymorphism of the angiotensin-converting-enzyme gene and left ventricular hypertrophy.* N Engl J Med, 1994. **331**(16): p. 1097-8.

17. Thomas, G.N., et al., *Renin-angiotensin system gene polymorphisms, blood pressure, dyslipidemia, and diabetes in Hong Kong Chinese: a significant association of tne ACE insertion/deletion polymorphism with type 2 diabetes.* Diabetes Care, 2001. **24**(2): p. 356-61.

18. Fan, X., et al., *Polymorphisms of ACE2 gene are associated with essential hypertension and antihypertensive effects of Captopril in women.* Clin Pharmacol Ther, 2007. **82**(2): p. 187-96.

19. Bautista, L.E., et al., *Population-based case-control study of renin-angiotensin system genes polymorphisms and hypertension among Hispanics.* Hypertens Res, 2008. **31**(3): p. 401-8.

20. Herrmann, F.H., et al., *Prevalence of eight molecular markers associated with thrombotic diseases in six Amerindian tribes and two African groups of Costa Rica.* Am J Hum Biol, 2004. **16**(1): p. 82-6.

21. Nadalin, S., et al., *The lack of association between angiotensin-converting enzyme gene insertion/deletion polymorphism and nicotine dependence in multiple sclerosis.* Brain Behav, 2017. **7**(1): p. e00600.

22. Bartakova, J., et al., *Association of the angiotensin I converting enzyme (ACE) gene polymorphisms with recurrent aphthous stomatitis in the Czech population: case-control study.* BMC Oral Health, 2022. **22**(1): p. 80.

23. Bladbjerg, E.M., et al., *Longevity is independent of common variations in genes associated with cardiovascular risk.* Thromb Haemost, 1999. **82**(3): p. 1100-5.

24. Taha, M., M.M.M. Ibrahim, and H. Sedrak, *Association of epistatic effects of MTHFR, ACE, APOB, and APOE gene polymorphisms with the risk of myocardial infarction and unstable angina in Egyptian patients.* Gene, 2024. **895**: p. 147976.

25. Boraey, N.F., et al., *Association of ACE1 I/D polymorphism and susceptibility to COVID-19 in Egyptian children and adolescents.* Pediatr Res, 2024.

26. AbdRaboh, N.R., et al., *Association of Angiotensin Converting Enzyme Insertion/Delition and Angiotensinogen T235 Polymorphisms with the Risk of Essential Hypertension in Egyptian Patients.* Int J Cancer Res, 2012. **8**(3): p. 69-82.

27. Maestu, J., et al., *Ace I/D polymorphism is associated with habitual physical activity in pubertal boys.* J Physiol Sci, 2013. **63**(6): p. 427-34.

28. Ash, G.I., et al., *No association between ACE gene variation and endurance athlete status in Ethiopians.* Med Sci Sports Exerc, 2011. **43**(4): p. 590-7.

29. Lahtela, E., et al., *ACE gene variants and sarcoidosis in a Finnish population.* Sarcoidosis Vasc Diffuse Lung Dis, 2017. **34**(2): p. 104-114.

30. Hadjadj, S., et al., *Prognostic value of the insertion/deletion polymorphism of the ACE gene in type 2 diabetic subjects: results from the Non-insulin-dependent Diabetes, Hypertension, Microalbuminuria or Proteinuria, Cardiovascular Events, and Ramipril (DIABHYCAR), Diabete de type 2, Nephropathie et Genetique (DIAB2NEPHROGENE), and Survie, Diabete de type 2 et Genetique (SURDIAGENE) studies.* Diabetes Care, 2008. **31**(9): p. 1847-52.

31. Ndong, A.G.R., et al., *Implication of insertion/deletion polymorphism of angiotensin converting enzyme gene in the occurrence of type 2 diabetes in the Gabonese Population.* Int J Biosciences, 2017. **11**(6): p. 61-67.

32. Filler, G., et al., *Renin angiotensin system gene polymorphisms in pediatric renal transplant recipients.* Pediatr Transplant, 2001. **5**(3): p. 166-73.

33. Ebert, M.P., et al., *The angiotensin I-converting enzyme gene insertion/deletion polymorphism is linked to early gastric cancer.* Cancer Epidemiol Biomarkers Prev, 2005. **14**(12): p. 2987-9.

34. Mohlendick, B., et al., *ACE2 polymorphism and susceptibility for SARS-CoV-2 infection and severity of COVID-19.* Pharmacogenet Genomics, 2021. **31**(8): p. 165-171.

35. Eleni, S., et al., *Angiotensin-I converting enzyme gene and I/D polymorphism distribution in the Greek population and a comparison with other European populations.* J Genet, 2008. **87**(1): p. 91-3.

36. Sgourou, A., et al., *Association of genome variations in the renin-angiotensin system with physical performance.* Hum Genomics, 2012. **6**(1): p. 24.

37. Kolovou, G., et al., *The frequency of 4 common gene polymorphisms in nonagenarians, centenarians, and average life span individuals.* Angiology, 2014. **65**(3): p. 210-5.

38. Barkai, L., A. Soos, and I. Vamosi, *Association of angiotensin-converting enzyme DD genotype with 24-h blood pressure abnormalities in normoalbuminuric children and adolescents with Type 1 diabetes.* Diabet Med, 2005. **22**(8): p. 1054-9.

39. Gupta, S., et al., *Angiotensin-converting enzyme gene polymorphism in hypertensive rural population of Haryana, India.* J Emerg Trauma Shock, 2009. **2**(3): p. 150-4.

40. Jhawat, V., et al., *Angiotensin Converting Enzyme Gene Insertion/Deletion Polymorphism Is Not Responsible for Antihypertensive Therapy Induced New Onset of Type 2 Diabetes in Essential Hypertension.* Clin Med Insights Endocrinol Diabetes, 2019. **12**: p. 1179551418825037.

41. Patel, D.D., et al., *Analysis of the Pattern, Alliance and Risk of rs1799752 (ACE I/D Polymorphism) with Essential Hypertension.* Indian J Clin Biochem, 2022. **37**(1): p. 18-28.

42. Bawazier, L.A., et al., *Relationship of angiotensin converting enzyme gene polymorphism and hypertension in Yogyakarta, Indonesia.* Acta Med Indones, 2010. **42**(4): p. 192-8.

43. Abbaszadeh, H., et al., *Angiotensin-converting enzyme 1 and voltage-gated potassium channel-interacting protein 4 gene polymorphisms in COVID-19 patients from east of Iran.* Clin Chim Acta, 2022. **536**: p. 39-44.

44. Panza, F., et al., *Lack of association between ace polymorphism and Alzheimer's disease in southern Italy.* Arch Gerontol Geriatr Suppl, 2002. **8**: p. 239-45.

45. Di Pasquale, P., et al., *Cardiovascular effects of I/D angiotensin-converting enzyme gene polymorphism in healthy subjects. Findings after follow-up of six years.* Acta Cardiol, 2005. **60**(4): p. 427-35.

46. Amir, O., et al., *The ACE deletion allele is associated with Israeli elite endurance athletes.* Exp Physiol, 2007. **92**(5): p. 881-6.

47. Kramer, H., et al., *Angiotensin-converting enzyme gene polymorphisms and obesity: an examination of three black populations.* Obes Res, 2005. **13**(5): p. 823-8.

48. Mannami, T., et al., *Low potentiality of angiotensin-converting enzyme gene insertion/deletion polymorphism as a useful predictive marker for carotid atherogenesis in a large general population of a Japanese city: the Suita study.* Stroke, 2001. **32**(6): p. 1250-6.

49. Wakai, K., et al., *Profile of participants and genotype distributions of 108 polymorphisms in a cross-sectional study of associations of genotypes with lifestyle and clinical factors: a project in the Japan Multi-Institutional Collaborative Cohort (J-MICC) Study.* J Epidemiol, 2011. **21**(3): p. 223-35.

50. AL-Eitan, L.N. and S.Z. Alahmad, *Allelic and genotypic analysis of the ACE I/D polymorphism for the possible prediction of COVID-19-related mortality and morbidity in Jordanian Arabs.* J Biosafety Biosecurity, 2023. **5**: p. 89-95.

51. Svyatova, G., et al., *Candidate genes related to acute cerebral circulatory disorders in Preeclampsia in the Kazakh Population.* J Stroke Cerebrovasc Dis, 2023. **32**(11): p. 107392.

52. Um, J.Y., et al., *Polymorphism of angiotensin-converting enzyme gene and BMI in obese Korean women.* Clin Chim Acta, 2003. **328**(1-2): p. 173-8.

53. Kwon, I., *Angiotensin-converting enzyme gene insertion/deletion polymorphism is not associated with BMI in Korean adults.* Phys Act Nutr, 2020. **24**(1): p. 24-28.

54. Khitrinskaya, I.Y., et al., *Genetic Differentiation of the Population of Central Asia Inferred from Autosomal Markers.* Russian J Gen, 2003. **39**(10): p. 1175–1183.

55. Paulauskas, A., et al., *Genetic variability associated with angiotensin converting enzyme (ace) gene polymorphism in sportsmen pursuing different sports.* Proc Latv Acad Sci, Sect. B, 2009. **63**(1/2): p. 9–13.

56. Saab, Y.B., P.R. Gard, and A.D. Overall, *The geographic distribution of the ACE II genotype: a novel finding.* Genet Res, 2007. **89**(4): p. 259-67.

57. Saad, H., et al., *The Role of Angiotensin Converting Enzyme 1 Insertion/Deletion Genetic Polymorphism in the Risk and Severity of COVID-19 Infection.* Front Med (Lausanne), 2021. **8**: p. 798571.

58. Wei, L.K., et al., *Clinical Relevance of MTHFR, eNOS, ACE, and ApoE Gene Polymorphisms and Serum Vitamin Profile among Malay Patients with Ischemic Stroke.* J Stroke Cerebrovasc Dis, 2015. **24**(9): p. 2017-25.

59. Thameem, F., et al., *Genetic polymorphisms in the renin-angiotensin system (RAS) genes and their association analysis with type 2 diabetes and related traits in Mexican Americans.* Diabetes Res Clin Pract, 2008. **79**(2): p. e14-6.

60. Capros, N., et al., *Aspects of the molecular-genetic profile in patients with ischemic heart disease.* Rev Med Chir Soc Med Nat Iasi, 2013. **117**(1): p. 78-82.

61. Comas, D., et al., *Alu insertion polymorphisms in NW Africa and the Iberian Peninsula: evidence for a strong genetic boundary through the Gibraltar Straits.* Hum Genet, 2000. **107**(4): p. 312-9.

62. van der Knaap, R., et al., *Renin-angiotensin system inhibitors, angiotensin I-converting enzyme gene insertion/deletion polymorphism, and cancer: the Rotterdam Study.* Cancer, 2008. **112**(4): p. 748-57.

63. Kooffreh, M.E., C.I. Anumudu, and P.L. Kumar, *Insertion/deletion polymorphism of the angiotensin-converting enzyme gene and the risk of hypertension among residents of two cities, South-South Nigeria.* Adv Biomed Res, 2014. **3**: p. 118.

64. Tronvik, E., et al., *Angiotensin-converting enzyme gene insertion/deletion polymorphism in migraine patients.* BMC Neurol, 2008. **8**: p. 4.

65. Mansoor, Q., et al., *Angiotensin-converting enzyme (ACE) gene II genotype protects against the development of diabetic peripheral neuropathy in type 2 diabetes mellitus.* J Diabetes, 2012. **4**(3): p. 257-61.

66. Oscanoa, T.J., et al., *Lack of association between angiotensin-converting enzyme genotype and muscle strength in Peruvian older people.* Adv Gerontol, 2020. **33**(4): p. 686-690.

67. Grandinetti, A., et al., *Association between angiotensin-converting enzyme gene polymorphisms and QT duration in a multiethnic population in Hawaii.* Auton Neurosci, 2006. **130**(1-2): p. 51-6.

68. Goracy, I., et al., *The Genetic Variants in the Renin-Angiotensin System and the Risk of Heart Failure in Polish Patients.* Genes (Basel), 2022. **13**(7).

69. Sousa, A.C., et al., *[Genetic Polymorphisms Associated with the Onset of Arterial Hypertension in a Portuguese Population].* Acta Med Port, 2018. **31**(10): p. 542-550.

70. Toma, M., et al., *Lack of association between ACE ID polymorphism and colorectal cancer in Romanian patients.* Chirurgia (Bucur), 2009. **104**(5): p. 553-6.

71. Procopciuc, L.M., et al., *Renin-angiotensin system gene variants and risk of early- and late-onset preeclampsia: A single center case-control study.* Pregnancy Hypertens, 2019. **18**: p. 1-8.

72. Gineviciene, V., et al., *Association analysis of ACE, ACTN3 and PPARGC1A gene polymorphisms in two cohorts of European strength and power athletes.* Biol Sport, 2016. **33**(3): p. 199-206.

73. Bondarenko, E.A., et al., *Genetic Analysis of BDNF, GNB3, MTHFR, ACE and APOE Variants in Major and Recurrent Depressive Disorders in Russia.* Int J Med Sci, 2016. **13**(12): p. 977-983.

74. Al-Harbi, K.M., et al., *Angiotensin-converting enzyme gene insertion/deletion polymorphism in Saudi patients with rheumatic heart disease.* Saudi Med J, 2015. **36**(2): p. 176-80.

75. Stankovic, M., et al., *Interactions of the eNOS and ACE genes and cigarette smoking in chronic obstructive pulmonary disease.* J Med Biochem, 2023. **42**(1): p. 94-104.

76. Sivakova, D., et al., *ACE insertion/deletion polymorphism and its relationships to the components of metabolic syndrome in elderly Slovaks.* Anthropol Anz, 2009. **67**(1): p. 1-11.

77. Alvarez, R., et al., *Angiotensin converting enzyme and endothelial nitric oxide synthase DNA polymorphisms and late onset Alzheimer's disease.* J Neurol Neurosurg Psychiatry, 1999. **67**(6): p. 733-6.

78. Romero-Blanco, C., et al., *Strength and Endurance Training in Older Women in Relation to ACTN3 R577X and ACE I/D Polymorphisms.* Int J Environ Res Public Health, 2020. **17**(4).

79. Bengtsson, K., et al., *Polymorphism in the angiotensin converting enzyme but not in the angiotensinogen gene is associated with hypertension and type 2 diabetes: the Skaraborg Hypertension and diabetes project.* J Hypertens, 1999. **17**(11): p. 1569-75.

80. Walder, B., et al., *Genetic heterogeneity in the renin-angiotensin system and the risk of diabetic nephropathy: Association with the angiotensinogen gene, but not with the ACE gene.* J Clin Bas Cardiol, 1998. **1**(1): p. 55-58.

81. Lee, Y.J. and J.C. Tsai, *ACE gene insertion/deletion polymorphism associated with 1998 World Health Organization definition of metabolic syndrome in Chinese type 2 diabetic patients.* Diabetes Care, 2002. **25**(6): p. 1002-8.

82. Chutinet, A., et al., *Association between genetic polymorphisms and sites of cervicocerebral artery atherosclerosis.* J Stroke Cerebrovasc Dis, 2012. **21**(5): p. 379-85.

83. Ezzidi, I., et al., *Identification of specific angiotensin-converting enzyme variants and haplotypes that confer risk and protection against type 2 diabetic nephropathy.* Diabetes Metab Res Rev, 2009. **25**(8): p. 717-24.

84. Mehri, S., et al., *Angiotensin-converting enzyme insertion/deletion gene polymorphism in a Tunisian healthy and acute myocardial infarction population.* Genet Test Mol Biomarkers, 2010. **14**(1): p. 85-91.

85. Bedir, A., et al., *Angiotensin converting enzyme gene polymorphism and activity in Turkish patients with essential hypertension.* Am J Hypertens, 1999. **12**(10 Pt 1): p. 1038-43.

86. Serdaroglu, E., et al., *ACE gene insertion/deletion polymorphism in childhood idiopathic nephrotic syndrome.* Pediatr Nephrol, 2005. **20**(12): p. 1738-43.

87. Drozdovska, S.B., et al., *The association of gene polymorphisms with athlete status in ukrainians.* Biol Sport, 2013. **30**(3): p. 163-7.

88. Kehoe, P.G., et al., *Variation in DCP1, encoding ACE, is associated with susceptibility to Alzheimer disease.* Nat Genet, 1999. **21**(1): p. 71-2.

89. Keavney, B., et al., *Large-scale test of hypothesised associations between the angiotensin-converting-enzyme insertion/deletion polymorphism and myocardial infarction in about 5000 cases and 6000 controls. International Studies of Infarct Survival (ISIS) Collaborators.* Lancet, 2000. **355**(9202): p. 434-42.

90. Steeds, R.P., et al., *Analysis of the postulated interaction between the angiotensin II sub-type 1 receptor gene A1166C polymorphism and the insertion/deletion polymorphism of the angiotensin converting enzyme gene on risk of myocardial infarction.* Atherosclerosis, 2001. **154**(1): p. 123-8.

91. Hidalgo, P.C., et al., *Questioning the "melting pot": analysis of Alu inserts in three population samples from Uruguay.* Hum Biol, 2014. **86**(2): p. 83-92.

92. Miners, S., et al., *Angiotensin-converting enzyme levels and activity in Alzheimer's disease: differences in brain and CSF ACE and association with ACE1 genotypes.* Am J Transl Res, 2009. **1**(2): p. 163-77.

93. Bakhtiyarova, G.K. and A.S. Babayeva, *I/D Polymorphism of the ACE Gene in Kazakh Origin Patients with Mitral Heart Disease.* Cukurova Med J, 2014. **39**(4): p. 848-854.

94. Kan, L.E., et al., *Features of Distribution I/D Polymorphous Marker of ace Gene in Patients of Uzbek Nationality with Unstable Angina and Coronary Heart Disease in Family History.* Liki Ukraini Plus, 2012. **3-4**(11-12): p. 33-36.

95. Itoyama, S., et al., *ACE1 polymorphism and progression of SARS.* Biochem Biophys Res Commun, 2004. **323**(3): p. 1124-9.
